# Characteristics of Soil Fungal Communities in Soybean Rotations

**DOI:** 10.3389/fpls.2022.926731

**Published:** 2022-06-23

**Authors:** Xiuli Song, Lei Huang, Yanqing Li, Chongzhao Zhao, Bo Tao, Wu Zhang

**Affiliations:** ^1^School of Geographical Sciences, Lingnan Normal University, Zhanjiang, China; ^2^Agricultural College, Northeast Agricultural University, Harbin, China

**Keywords:** continuous soybean cropping, rotation, fungal community structure and diversity, potential pathogenic genera, soil chemical properties

## Abstract

Soybean continuous cropping (SC) leads to continuous cropping obstacles, and soil-borne fungal diseases occur frequently. Rotation can alleviate continuous cropping obstacles. However, the long-term effects of continuous cropping and rotation on the structure and function of the fungal community in soil are not clear. In this study, five cropping systems, SC, fallow (CK), fallow-soybean (FS), corn–soybean (CS), and wheat–soybean (WS), were implemented in the long-term continuous cropping area of soybean. After 13 years of planting, high-throughput sequencing was used to evaluate the structure and diversity of soil fungal communities and to study the relationship between fungal communities and soil environmental factors. The results showed that the abundance and diversity of fungal flora in SC soil were the highest. There were significant differences in the formation of soil fungal communities between soybean continuous cropping and the other treatments. There were 355 species of endemic fungi in SC soil. There were 231 and 120 endemic species in WS and CS, respectively. The relative abundance of the potential pathogens *Lectera*, *Gibberella*, and *Fusarium* in the SC treatment soil was significantly high, and the abundance of all potential pathogens in CK was significantly the lowest. The abundance of *Lectera* and *Fusarium* in CS was significantly the lowest. There was a positive correlation between potential pathogens in the soil. The relative abundance of potential pathogens in the soil was significantly positively correlated with the relative abundance of Ascomycetes and negatively correlated with the relative abundance of Basidiomycetes. Potential pathogenic genera had a significant negative correlation with soil OM, available Mn, K and soil pH and a significant positive correlation with the contents of soil available Cu, Fe, and Zn. In general, the fungal communities of SC, FS, WS, and CS were divided into one group, which was significantly different from CK. WS and CS were more similar in fungal community structure. The CK and CS treatments reduced the relative abundance of soil fungi and potential pathogens. Our study shows that SC and FS lead to selective stress on fungi and pathogenic fungi and lead to the development of fungal community abundance and diversity, while CK and CS can reduce this development, which is conducive to plant health.

## Introduction

China has a great demand for soybeans (Liu et al., 2020). Northeast China is not only an important production base for food security but also a major soybean production area in China. For economic benefit, some farms in Northeast China have been planting soybeans for more than 40 consecutive years (Liu et al., 2020). Continuous planting of soybean leads to continuous cropping obstacles in soil (Liu et al., 2012; Benitez et al., 2017) and aggravation of soil-borne diseases (Yan et al., 2012; Pérez-Brandán et al., 2014; Bai et al., 2015). Studies have shown that these changes are closely related to biological factors in soil (Dias et al., 2015; Liu et al., 2020).

Soil microorganisms play an important role in maintaining ecosystem sustainability and plant health (Avidano et al., 2005). Different cropping systems can significantly change the microbial community structure (Zhou et al., 2018; Liu H. et al., 2019). However, these changes depend on the type of cropping system, soil type and crop type. Xiong et al. (2016) found that continuous cropping significantly increased the proportion of fungi in soil. Rotation can significantly reduce the number of fungal populations (Yu et al., 2011), facilitate the reproduction of plant growth promotion in soil (Baetz and Martinoia, 2014; Pii et al., 2015), and enhance the stability and resilience of soil ecosystems (Clemente et al., 2005; Behera et al., 2007). Understanding how different cropping systems affect the composition of the fungal microbial community is conducive to the regulation of the microbial community through planting and the prevention and treatment of soil-borne diseases in agricultural production.

Soybean continuous cropping (SC) increases the abundance of soil pathogenic fungi and induces crop diseases (Guo et al., 2011; Wang et al., 2013). The change in soil fungal community structure in SC is mainly driven by 14 genera, and *Guehomyces*, *Alternaria*, and *Metacordyceps* contribute more to the change in the fungal community (Liu H. et al., 2019). The pathogenic *Fusarium* sp. in soil is the main pathogen of soybean root rot, which can cause soybean plant wilt at all stages of soybean growth (Liu J. et al., 2019). Crop rotation is an agronomic practice adopted by farmers after the serious outbreak of soil-borne diseases, and it has been proven that they can be successfully controlled (Wright et al., 2015). Studies have shown that crop rotation can maintain the diversity of soil microbial communities and enhance disease inhibition (Peralta et al., 2018). Wheat soybean rotation can reduce the number of *Fusarium* in wheat soil, which may effectively reduce the risk of wheat diseases (Lu et al., 2022). The research results are inconsistent due to the different soil types, rotation systems and continuous cropping years. The related mechanism of continuous cropping obstacles is complex and needs to be explored in many aspects under different conditions.

Soil physical and chemical properties affect the structure and function of the soil microbial community (Xiang et al., 2020). Prior studies have shown that soil total phosphorus and available phosphorus similarly influence the soil fungal community structure and the relative abundance of specific fungi (Yao et al., 2019). Increasing soil organic matter can inhibit fungal pathogens (Saxena et al., 2015). Basic trace elements (TE), such as Zn, Fe, Mn, and Cu, are the main cofactors of various proteins in biological systems and have important biological significance (Pilon et al., 2009). As reported, the fungal community was most correlated with available P, K, and Cu (Song et al., 2018). There have been few studies on the correlation between soil mineral elements and the soil microbial community (Cai et al., 2015).

Long-term unreasonable cultivation makes the soil quality and microecology unbalanced, and crop diseases occur frequently, which seriously affects the quality of crop products and the sustainable development and utilization of cold black soil. Previous studies have mainly focused on the effects of agronomy, cultivation, soil type, fertilizer, and heavy metals on the overall number and diversity of microbial communities or on a certain group of microorganisms under long-term positioning tests (Liu et al., 2010; Hartmann et al., 2015; Li et al., 2018). However, it is not clear how the implementation of rotation cropping systems under long-term continuous cropping will change the structure and function of the fungal community in continuous cropping soil. In this study, the effects of fallow, fallow-soybean (FS), corn–soybean (CS), and wheat–soybean (WS) rotations on the soil fungal community of soybean continuous cropping were studied, and the relationship between cropping systems, soil mineral elements and fungal community structure was explored. We focused on the effects of different cropping systems on harmful and beneficial microbial communities in soil fungal communities. The results enable us to have a clearer understanding of the diversity and composition structure of soil fungal communities under different rotation systems to provide a theoretical basis for formulating an effective rotation cropping system.

## Materials and Methods

### Study Site Description and Soil Sample Collection

The test site is located in Nenjiang County, Heilongjiang Province, China (124°68′ E, 49° N). The soil type belongs to the Vertisol class according to the American soil classification system (Soil Survey Staff, 2014). The annual average temperature is 0.8∼1.4°C, the annual cumulative temperature (greater than or equal to 10°C) is approximately 2,230°C, and the annual rainfall is approximately 480–512 mm. Before the experiment, the experimental site was a soybean continuous cropping area. The long-term positioning experiment was set up in April 2005. The experiment was set up as five treatments: fallow (CK), SC, FS rotation, CS rotation, and WS rotation. Three replications were set for each treatment, and the area of each plot was 666.7 m^2^. A randomized block arrangement was used. In the year of the cropping season, wheat was sown from April 1 to April 10, corn was sown from April 30 to May 10, and soybeans were sown from May 10 to May 20. Urea, diammonium phosphate and potassium sulfate were applied once before crop planting. There were 33.1 kg N ha^–1^, 24.1 kg P ha^–1^ and 21.6 kg K ha^–1^ applied for soybean, 59.0 kg N ha^–1^, 40.1 kg P ha^–1^ and 43.1 kg K ha^–1^ for maize, and 73.0 kg N ha^–1^, 30.1 kg P ha^–1^ and 21.6 kg K ha^–1^ for wheat. An addition of 59.0 kg N ha^–1^ was applied at the booting stage of maize. Wheat was harvested from August 1 to August 10, soybeans were harvested from September 20 to October 5, and corn as harvested from October 10 to October 30. On October 30, 2017, soil samples were collected after soybean harvest. According to the aseptic requirements, for each plot, 10 surface soil samples with a depth of 0–20 cm were randomly taken with a soil auger with a diameter of 5 cm and put into a sterile bag for full mixing. This process was repeated three times, and the samples were mixed into one soil sample. A total of 15 soil samples were collected from the five treatments. After returning to the laboratory, the excess parts were removed, and stones, plant roots and animal residues were removed and screened through a 2 mm sterile sieve. Some soil samples in each soil sample were placed in sterilized microcentrifuge tubes and stored at −80°C for DNA extraction. The remaining soil was air dried to measure the chemical properties of the soil.

### Analysis of Physical and Chemical Properties of Soil Samples

Soil samples were dried at room temperature and ground into pieces that passed through a 2 mm sieve. The soil micronutrients Fe, Mn, Zn, and Cu were extracted with diethylenetriaminepentaacetic acid followed by atomic absorption spectrophotometry analysis (Lindsay and Norvell, 1978). The available B was determined by potassium imine colorimetry (Malekani and Cresser, 1998), and the pH was measured at a soil-to-water ratio of 2.5:1. Soil organic C was determined following wet digestion (Walkley and Black, 1934), and the values were multiplied by a factor of 1.724 to obtain organic matter (OM) values. A basic N solution was created using the diffusion method (Bremner and Keeney, 1966), and available P was determined using the 0.5 mol⋅L^–1^ NaHCO_3_ leaching-molybdenum antimony colorimetric method (Föhse et al., 1991). The available potassium (K) was measured with the 1 mol L^–1^ NH_4_OAc extraction-flame photometric method (Petersen and Corey, 1966).

### Soil DNA Extraction

Microbial DNA was extracted from 0.5 g samples using an E.Z.N.A.^®^ soil DNA Kit (Omega Bio-Tek, Norcross, GA, United States) according to the manufacturer’s instructions. The final DNA concentration and purification were determined using a NanoDrop 2000 ultraviolet–visible (UV–Vis) spectrophotometer (Thermo Scientific, Wilmington, DE, United States), and the DNA quality was checked using 1% agarose gel electrophoresis.

### Quantitative Real-Time PCR

The fungal internal transcribed spacer (ITS) region of the rRNA gene copy number for all samples was determined in triplicate using Q-PCR in an ABI 7500 Real-Time PCR System (Applied Biosystems, Carlsbad, CA, United States) with the primer set ITS1F/ITS2R (Adams et al., 2013). Each PCR contained 16.5 μL of AceQ^®^ RSYBR Green quantitative real-time PCR (qPCR) Master Mix (2X), 0.8 μL of 5 μM forward and reverse primers (each) and 2.0 μL of template DNA. The PCR samples were subsequently incubated at 95°C for 5 min, followed by 40 cycles of 5 s at 95°C, 30 s for annealing at 50°C, and 40 s for elongation at 72°C. Negative controls consisted of all the reagents with sterilized water instead of soil DNA. The threshold cycle (Ct) was obtained from triplicate samples and averaged. The copy number of the fungal ITS genes was calculated using a regression equation to convert the cycle threshold (Ct) value to the known number of copies in the standard curves.

### Illumina MiSeq Sequencing

The ITS rRNA genes were amplified using the primers ITS1F (5′-CTTGGTCATTTAGAGGAAGTAA-3′) and ITS2R (5′-GCTGCGTTCTTCATCGATGC-3′) (Adams et al., 2013) in a thermocycler PCR system (GeneAmp 9700, ABI, Foster, CA, United States). The PCRs were conducted using the following program: 3 min of denaturation at 95°C; 35 cycles of 30 s at 95°C, 30 s for annealing at 55°C, and 45 s for elongation at 72°C; and a final extension at 72°C for 10 min. The PCR products were purified and then sequenced using the MiSeq Illumina platform (Illumina, United States) at Genesky Biotechnologies Inc. (Shanghai, China). The raw reads were deposited into the National Center for Biotechnology Information (NCBI) Sequence Read Archive (SRA) database (accession number: SRP164820).

### Processing of Sequencing Data

After sequencing, the raw FASTQ files were processed using QIIME Pipeline Version 1.19.1. Briefly, all sequence reads were assigned to each sample based on the barcodes. Sequences with low quality (length < 200 bp and average base quality score < 20) were removed before further analysis. The chimeras of trimmed sequences were detected and removed using the UCHIME algorithm (Edgar et al., 2011). After filtering the data, the quality value of the reassembled base Q20 is greater than 99%, and Q30 is greater than 96%. These results fully met the requirements of subsequent data analysis (Supplementary Tables 1, 2). The sequences were phylogenetically assigned according to their best matches to sequences in the Ribosomal Database Project (RDP) database using the RDP classifier (Cole et al., 2009). Operational taxonomic units (OTUs) were classified at 97% sequence similarity using CD-HIT (Li and Godzik, 2015). We rarified the abundance matrix to 110737 (the lowest sequence read depth across the study) sequences per sample to obtain normalized relative abundances. Chao1 richness and Shannon’s diversity index were calculated in QIIME. Constrained principal coordinate analysis (CPCoA) and significance tests (Adonis test and Mantel test) were performed in R version 3.5.1 for Windows with the “vegan” package. Mothur (version 1.33.3) was used for alpha diversity analysis [species richness statistics, such as Chao and the abundance-based coverage estimator (ACE), and species diversity statistics, such as Shannon and Simpson], Venn diagrams and beta diversity analysis (PCA). Linear discriminant analysis (LDA) effect size (LEfSe) was used to elucidate the biomarkers in each treatment. Those with an LDA score ≥ 2.0 were considered to be important biomarkers in each treatment (Xiang et al., 2020). The cladogram was drawn using the Huttenhower Galaxy web application *via* the LEfSe algorithm (Segata et al., 2011).

### Statistical Analysis

The species with significant abundance differences among different groups were detected by the non-parametric Kruskal–Wallis rank sum test. Statistical analyses were performed, and heatmap analysis was performed using the multcomp package in R (3.4.1) (R Development Core Team, 2010) to reveal significant differences in the fungal genera. Additionally, we predicted potentially pathogenic and beneficial fungi using FUNGuild^5^ (Nguyen et al., 2016). The pathogenic and beneficial fungi were assigned according to their potential to damage or benefit the plant. The stepwise analysis and correlation test of biological environmental connections were carried out in R (3.4.1) (R Development Core Team, 2010), and the environmental factor with the highest correlation with the community (OTU) was calculated and counted. Pearson correlation analysis was used to test the significance of correlations between fungi. The species with significant abundance differences among different groups were detected by the non-parametric Kruskal–Wallis rank sum test.

## Results

### Effects of Cropping Systems on the Soil Fungal Community

**FIGURE 1:**
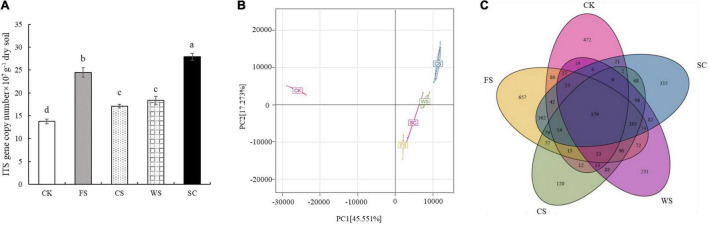
Differences in soil fungal community structure in different cropping systems. **(A)** The abundance of fungal ITS rRNA gene copies assessed by real-time PCR in five soybean cropping systems. Different letters represent significant differences (*P* < 0.05), **(B)** principal coordinate analysis (PCA) plot based on the OTUs for five soybean cropping systems. **(C)** Venn diagram of OTU distribution comparison. The numbers represent the number of species. SC, soybean continuous cropping; CK, fallow; FS, fallow-soybean; CS, corn–soybean; WS, wheat–soybean.

**FIGURE 2:**
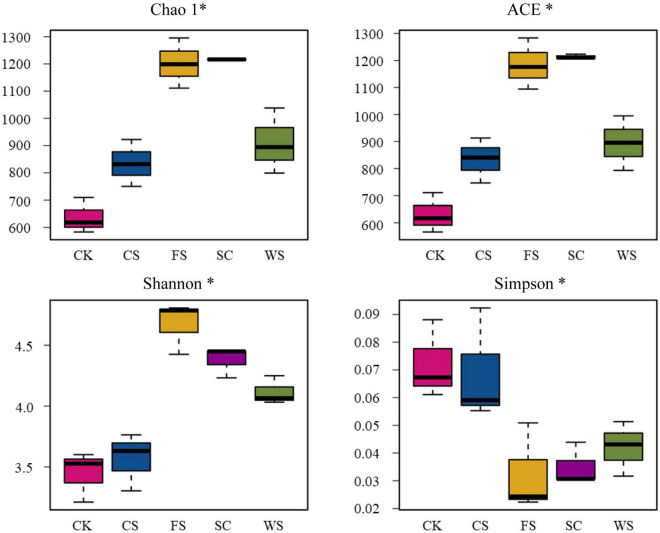
Chao1 index, ACE index, Shannon index, and Simpson index for fungi in five soybean cropping systems with > 97% sequence identity. *Indicates a significant correlation (*P* < 0.05).

The number of fungi was quantified by real-time PCR. Compared with CK, SC, FS, WS, and CS significantly increased the abundance of fungi, which were 2.02, 1.78, 1.33, and 1.24 times that of CK, respectively (Figure 1A). The PCoA plot and cluster analysis based on the OTU analysis showed clear similarities or differences among the fungal community structures across all the samples. All of the soil samples were separated into two groups. The fungal communities in FS, SC, WS, and CS were grouped together and were clearly separated from CK. WS and CS were grouped together, indicating that WS and CS had similar fungal community structures on PCA (Figure 1B and Supplementary Figure 1). Venn diagram analysis shows that the number of endemic fungal species in FS planting soil was the largest, 657 species. A total of 472 species of endemic fungi were formed in CK soil. There are 355 species of endemic fungi in SC soil. There were 231 and 120 endemic species in WS and CS, respectively (Figure 1C). Continuous cropping of FS and SC increased the fungal community diversity. The Shannon index was significantly higher in FS and SC than in CK and CS. The Chao1 and ACE indexes demonstrated that the upper limits of FS and SC were significantly higher than those of CK and CS. The relative abundance and diversity of fungi in CK soil were the lowest (Figure 2). Compared with SC, the abundance and diversity of fungi in CS and WS soil decreased significantly, and CS treatment was the lowest.

### Effects of Cropping Systems on the Floristic Composition of the Soil Fungal Community

**FIGURE 3:**
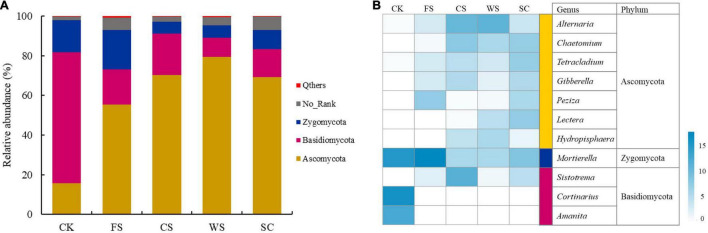
Relative abundance of different fungi at the phylum level **(A)** and heatmap analysis **(B)** of the dominant fungal genera with relative abundance greater than 0.5% in five soybean cropping systems. The color code indicates relative abundance, ranging from white (low abundance) to blue (high abundance).

The soil fungi in the different treatments were mainly distributed in Ascomycota, Basidiomycota and Zygomycota (Figure 3A and Supplementary Table 3). The relative abundance of Ascomycota was significantly higher in WS, followed by CS, SC, FS, and CK (*p* < 0.05). The relative abundance of Basidiomycota was significantly higher in CK, followed by CS, FS, SC, and WS (*p* < 0.05). The relative abundance of Zygomycota was significantly higher in FS, followed by CK, SC, WS, and CS (*p* < 0.05).

The relative abundance of *Alternaria* in Ascomycota was significantly higher in CS and WS. The relative abundance of *Chaetomium* was significantly higher in CS, WS, and SC. The relative abundance of *Tetraclaudium* was significantly higher in SC. The relative abundance of *Gibberella* was significantly higher in SC and CS. The relative abundance of *Peziza* was significantly high in FS (Figure 3B and Supplementary Table 4). The relative abundance of *Mortierella* in Zygomycota was significantly higher in FS, followed by CK and SC. The relative abundance of *Cortinarius* and *Amanita* in Basidiomycota was significantly higher in CK. The relative abundance of *Sistotrema* was significantly higher in CS (Figure 3B and Supplementary Table 4).

**FIGURE 4:**
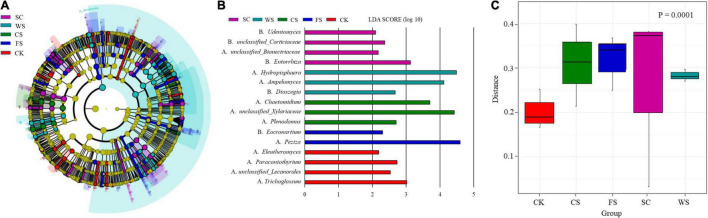
Analysis of species and community functions of fungi with significant differences in soil under different cropping systems. Different color areas represent different groups. **(A)** Taxonomic cladogram produced from LEfSe. The phylum, class, order, family, and genus levels are listed in order from inside to outside of the cladogram. LEfSe diagram. The corresponding color nodes in the diagram represent the groups that play an important role in this grouping (significantly different from other groups), and the yellow nodes represent the microbial groups that do not play an important role. **(B)** Shows the species with significant differences in abundance in different groups, and the length of the histogram represents the influence degree of the species with significant differences. **(C)** The UniFrac distance matrix was used to draw the distance diagram between sample groups. The ordinate represents the sample weighted UniFrac distance, and the *p*-value in the upper right corner represents the test results of the difference between groups.

Fungal groups significantly different from other treatments were formed in the soils of different cropping systems (Figure 4A). Different fungal groups have an important impact on the division of fungal communities in different cropping systems. The relative abundances of *Trichoglossum*, *Paraconiothyrium* and *Unclassified_Lecanorales* and *Eleutheromyces* were more abundant following CK amendment. The relative abundances of *Entorrhiza*, *Unclassified_Corticiaceae*, *Unclassified_Bionectriaceae* and *Udeniomyces* were more abundant following SC amendment. The relative abundances of *Unclassified_Xylariaceae*, *Chaetomidium*, and *Plenodomus* were more abundant following CS amendment. The relative abundances of *Hydropisphaera*, *Ampelomyces*, and *Dioszegia* were higher following WS amendment. The relative abundances of *Eocronartium* and *Peziza* were higher following FS amendment (Figure 4B).

The formation of fungal community function was significantly different in different treatments (Figure 4C). Compared with SC, the relative abundance of *Cryptococcus* in CK exhibited an extremely significant decline, the relative abundance of T*etraclaudium*, *Alternaria*, *Clonostachys*, and *Chaetomium* decreased significantly, and the relative abundance of *Pseudonymnoascus* increased significantly (Supplementary Figure 2). Compared with SC, the relative abundance of *Sarocladium* in FS soil increased significantly, *Vishniacozyma*, *Fusicolla*, *Pseudonymnoascus*, *Solicoccozyma*, *Peziza*, *Paraglomus*, and *Penicillium* increased significantly, and the relative abundance of *Cryptococcus*, *Cladosporium*, *Mrakia*, *Cryptococcus*, *CladosporiumMrakia*, and *Tetraclaudium* decreased significantly. The relative abundance of *Tetraclaudium, Hydropisphaera, Clonostachys, Chaetomium, Metarhizonium, Acremonium*, and *Atractospora* decreased significantly (Supplementary Figure 3). Compared with SC, the relative abundance of *Chaetomidium*, *Nectria*, and *Alternaria* in CS increased significantly, and the relative abundance of *Cladosporium*, *Peziza*, and *Cryptococcus* decreased significantly. The relative abundance of *Mrakia*, *Metarhizonium*, *Clonostachys*, *Nectria*, and *Exophiala* decreased significantly (Supplementary Figure 4). Compared with SC, the relative abundance of *Nectria* in WS increased significantly, the relative abundance of *Hydropisphaera*, *Guehomyces*, *Alternaria*, *Humicola, Periconia, Pseudonymnoascus*, and *Atractospora* increased significantly, the relative abundance of *Peziza* and *Cladosporium* decreased significantly, and the relative abundance of *Tetracladium, Moraia, Metarhizium, Exophiala, Unclassified_Halosphaeriaeae*, and *Herbotrichia* decreased significantly (Supplementary Figure 5).

### Potential Pathogenic and Beneficial Fungi

The relative abundances of potential pathogenic *Lectera, Gibberella, and Fusarium* in SC soil were significantly high. The relative abundances of *Fusarium, Sarocladium*, and *Volutella* in FS soil were significantly high. The relative abundances of *Alternaria*, *Lectera*, *Fusarium*, *Boeremia*, *Dendyphion*, *Volutella*, and *Bipolaris* in WS soil were significantly high. The relative abundances of *Alternaria*, *Gibberella*, and *Volutella* in CS soil were significantly high. The relative abundance of all potential pathogens in CK soil was significantly the lowest (Supplementary Figure 6A).

The relative abundance of the beneficial fungus *Penicillium* was significantly high in CK. The relative abundances of *Mortierella* and *Penicillium* in FS were significantly higher, and the relative abundance of *Chaetomium* in CS was significantly higher. The relative abundances of *Chaetomium*, *Cryptococcus*, and *Clonostachys* in WS were significantly high. The relative abundances of *Chaetomium*, *Cryptococcus*, *Metarhizonium*, *Acremonium*, and *Hirsutella* in SC were significantly high (Supplementary Figure 6B).

**FIGURE 5:**
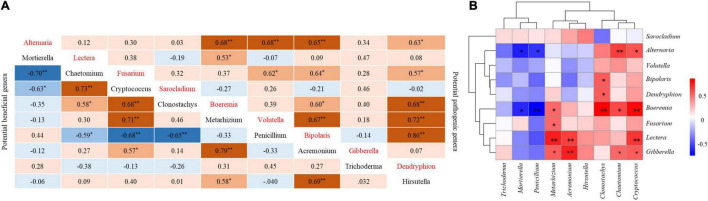
Correlation between potential pathogenic genera in soil and potentially beneficial genera. **(A)** Correlation between potential pathogenic genera and pathogenic genera and between potential beneficial genera and beneficial genera. **(B)** Correlation between potential pathogenic genera and beneficial genera. *Indicates a significant correlation (*P* < 0.05), ^**^indicates an extremely significant correlation (*P* < 0.01).

There was a significant positive correlation between potential pathogen genera and pathogen genera in soil, and *Alternaria* was very significantly positively correlated with *Boeremia*, *Volutella*, and *Bipolaris*. There was a very significant positive correlation between *Volutella* and *Bipolaris*. *Dendryphion* was significantly positively correlated with *Bipolaris, Volutella, and Boeremia* (Figure 5A).

*Mortierella* was very negatively correlated with *Chaetomium* in beneficial fungi. *Penicillium* was very negatively correlated with *Cryptococcus* and *Clonostachys*. *Chaetomium* was very positively correlated with *Cryptococcus*. *Cryptococcus* was positively correlated with *Clonostachys* and *Metarhizoum*. There was a very significant positive correlation between *Metarhizoum* and *Acremonium*. *Acremonium* was positively correlated with *Hirsutella* (Figure 5A).

The beneficial fungus *Mortierella* was significantly negatively correlated with the pathogenic fungi *Alternaria* and *Boeremia*. There was a significant negative correlation between the beneficial fungus *Penicillium* and the pathogenic fungi *Alternaria* and *Boeremi*. Among the beneficial fungi, the relative abundance of *Chaetomium*, *Cryptococcus*, *Clonostachys*, *Acremonium*, and *Metarhizobium* had a significant positive correlation with some pathogens (Figure 5B).

The relative abundance of *Fusarium* was significantly positively correlated with the Chao 1 and Shannon indexes of soil fungi, and the correlation coefficients were 0.585 and 0.619, respectively (*p* < 0.05). The relative abundance of *Sarocladium* was significantly positively correlated with the Chao 1 and Shannon indexes of soil fungi, and the correlation coefficients were 0.664 and 0.536, respectively (*p* < 0.05). Other potential pathogens had no significant correlation with the abundance and diversity of soil fungi. The relative abundances of *Alternaria*, *Fusarium*, *Boeremia*, *Volutella*, *Bipolaris*, *Gibberella*, and *Denryphion* were significantly positively correlated with Ascomycota abundances, and the correlation coefficients were 0.759, 0.615, 0.777, 0.684, 0.528, 0.649, and 0.682, respectively (*p* < 0.05). The relative abundances of *Alternaria*, *Fusarium*, *Boeremia*, *Volutella*, *Bipolaris*, *Gibberella*, and *Denryphion* were significantly negatively correlated with the abundances of Basidiomycota, and the correlation coefficients were −0.592, −0.787, −0.655, −0.655, −0.529, −0.637, and −0.615, respectively (*p* < 0.05). The relative abundances of *Alternaria* and *Boeremia* were significantly negatively correlated with the abundance of Zygomycota, and the correlation coefficients were −0.645 and −0.613, respectively (*p* < 0.05). The relative abundance of *Metarhizobium* was positively correlated with the abundance and diversity of the Chao 1 and Shannon indexes (*p* < 0.01). The relative abundance of *Hirsutella* was significantly positively correlated with the Chao 1 and Shannon indexes of fungi (*p* < 0.05). The relative abundance of *Acremonium* was significantly positively correlated with the Chao 1 indexes of fungi (*p* < 0.05). There was no significant correlation between other fungal abundance and diversity. The relative abundance of *Chaetomium*, *Cryptococcus*, *Clonostachys*, and *Metarhizobium* was significantly positively correlated with the abundance of Ascomycota and negatively correlated with the abundance of Basidiomycota (*p* < 0.05). *Mortierella* relative abundance was significantly negatively correlated with Ascomycota abundance and positively correlated with Zygomycota abundance (*p* < 0.05). The relative abundance of *Penicillium* was significantly negatively correlated with the abundance of Ascomycota (*p* < 0.05). The relative abundance of *Chaetomium* and *Cryptococcus* was significantly negatively correlated with the abundance of Zygomycota (*p* < 0.05) (Table 1).

**TABLE 1:** Correlations between potential pathogenic genera, potentially beneficial genera and the fungal community.

	Fungal diversity				Dominant fungi phylum		
	Chao1	ACE	Shannon	Simpson	Ascomycota	Basidiomycota	Zygomycota
**Potential pathogenic genera**							
*Alternaria*	–0.021	–0.025	–0.068	–0.051	0.759**	−0.592*	−0.645**
*Lectera*	0.300	0.305	0.325	–0.427	0.482	–0.465	–0.281
*Fusarium*	0.585*	0.574*	0.619*	–0.508	0.615*	−0.787**	0.181
*Sarocladium*	0.664**	0.668**	0.536*	–0.398	0.273	–0.415	0.219
*Boeremia*	0.225	0.218	0.150	–0.268	0.777**	−0.655**	−0.613*
*Volutella*	0.204	0.206	0.281	–0.279	0.684**	−0.655**	–0.293
*Bipolaris*	0.034	0.009	0.166	–0.160	0.528*	−0.529*	–0.162
*Gibberella*	0.510	0.521*	0.306	–0.344	0.649**	−0.637*	–0.284
*Dendryphion*	0.268	0.243	0.373	–0.401	0.628*	−0.615*	–0.259
Potential beneficial genera							
*Mortierella*	0.112	0.097	0.175	–0.030	−0.555*	0.250	0.998**
*Chaetomium*	0.154	0.181	–0.020	–0.082	0.716**	−0.549*	−0.701**
*Cryptococcus*	0.453	0.456	0.315	–0.406	0.837**	−0.737**	−0.622*
*Clonostachys*	0.203	0.202	0.150	–0.267	0.574*	−0.521*	–0.365
*Metarhizium*	0.803**	0.805**	0.722**	−0.713**	0.573*	−0.663**	–0.102
*Penicillium*	–0.069	–0.070	0.169	–0.132	−0.569*	0.467	0.456
*Acremonium*	0.630*	0.638*	0.445	–0.452	0.393	–0.448	–0.091
*Trichoderma*	0.338	0.318	0.349	–0.295	–0.135	0.019	0.311
*Hirsutella*	0.616*	0.617*	0.675**	−0.639*	0.363	–0.451	–0.029

*Indicates a significant correlation (P < 0.05), **indicates an extremely significant correlation (P < 0.01).

### Correlation Between Soil Fungi and Soil Chemical Properties

**FIGURE 6:**
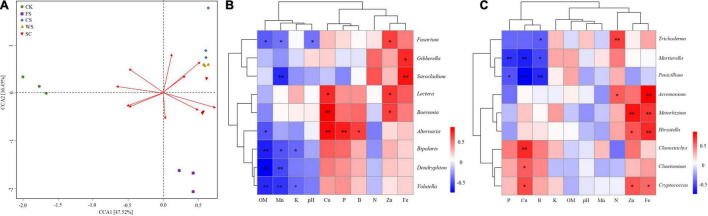
Correlation between soil fungi and soil chemical properties. **(A)** RDA diagram of soil environmental factors and fungi. Arrow rays represent different environmental factors. The longer the ray is, the greater the impact of the environmental factor. The included angle between the sample fungal community central line and the arrow represents the correlation between the fungal and environmental factors (an acute angle indicates a positive correlation, an obtuse angle indicates a negative correlation, and a right angle indicates no correlation); points of different colors represent different samples; **(B)** potentially potential pathogen; **(C)** potentially beneficial fungi. *Indicates a significant correlation (*P* < 0.05), **indicates an extremely significant correlation (*P* < 0.01).

There were significant differences in the correlations between different chemical properties in the soil and soil fungal communities, among which the contents of available Cu, Fe, and Mn were highly correlated with the fungal community (Figure 6A and Supplementary Table 5). Ascomycota had a very significant positive correlation with the effective content of Cu and a significant negative correlation with the effective content of OM, Mn, and Fe. Basidiomycota had a significant negative correlation with the effective contents of Zn and Fe and a significant positive correlation with the effective content of Mn. Zygomycota was significantly negatively correlated with the effective contents of Cu and P (Table 2). The potential pathogens were mainly negatively correlated with the effective contents of OM, Mn, K, and soil pH and positively correlated with the effective contents of Cu, Fe and Zn.

**TABLE 2:** Correlation coefficients between the main microbial communities (phylum level) and soil physical and chemical properties.

	OM	N	P	K	PH	Cu	Zn	Mn	Fe
Ascomycota	−0.60*	–	–	–	–	0.68**	–	−0.55*	0.58*
Basidiomycota	–	–	–	–	–	–	−0.57*	0.59*	−0.68**
Zygomycota	–	–	−0.64**	–	–	−0.71*	–	–	–

*Indicates a significant correlation (P < 0.05), ** indicates an extremely significant correlation (P < 0.01).

*Fusarium* was significantly negatively correlated with soil OM, available Mn content and soil pH. *Sarocladium* was very significantly negatively correlated with the effective content of Mn. *Bipolaris, Dendyphion*, and *Volutella* were negatively correlated with OM content. *Bipolaris* was significantly negatively correlated with the effective contents of Mn and K. *Dendriphion*, *Volutella*, and Mn effective content were significantly negatively correlated. *Volutella* was negatively correlated with the effective content of K. *Alternaria* was positively correlated with the effective contents of Cu and P.

*Boeremia* was positively correlated with the effective content of Cu. *Sarocladium* was very significantly positively correlated with the effective content of Fe. *Fusarium*, *Lectera*, and *Boeremia* were significantly positively correlated with the effective content of Zn (Figure 6B). The potential beneficial fungus *Trichoderma* was positively correlated with the effective content of N. There was a very significant positive correlation between *Metarhizoum* and the effective contents of Fe and Zn. *Acremonium* and *Hirsutella* were significantly positively correlated with the effective content of Fe. *Clonostachys* was significantly positively correlated with the effective content of Cu. *Mortierella* was negatively correlated with the effective contents of P and Cu. *Penicillium* was significantly negatively correlated with the content of available Cu and B (Figure 6C).

## Discussion

### Effects of Cropping Systems on Fungal Abundance and Community Composition

The species diversity and community structure of soil microorganisms determine the ability of soil to resist pathogens, which is very important for the sustainable development of soil ecosystems (Smith et al., 2008). Continuous cropping will cause an imbalance of soil, a surge of pathogens and an increased probability of crop disease (Meriles et al., 2009). In this study, it was confirmed that there were significant differences in the formation of fungal communities in the soil of the five cropping systems. All of the soil samples were separated into two groups. The fungal communities in FS, SC, WS, and CS were grouped together and were clearly separated from CK, and the similarity of the WS and CS fungal communities was the highest (Figure 1A). The soil fungal abundance (Chao1/ACE) and fungal diversity (Shannon) were significantly the highest in FS and SC (Figure 2). The relative abundance of Ascomycota in SC was high, and the relative abundance of unknown fungal flora increased significantly (relative abundance of no rank), which may be because SC significantly increased the number of endemic fungi (Supplementary Table 3). This study confirmed that SC could increase the relative abundance and diversity of soil fungi. Studies have shown that soybean roots secrete a large number of flavonoids and phenolic acids (Guo et al., 2011), and flavonoids and phenolic acids have a significant impact on fungal pathogens (Wang et al., 2013). Compared with SC, CK, and CS significantly reduced the number and diversity of fungi, and the abundance and diversity of fungi in CK were significantly the lowest. Other studies have shown that there is no difference in the diversity of fungal communities between continuous planting and corresponding rotation systems (Li et al., 2010; Liu et al., 2014a). Research has also shown that soybean maize rotation increases the diversity of fungal communities in rhizosphere and massive soil (Liu J. et al., 2019). This is inconsistent with the results of this study and may be caused by different soil environments and planting management systems. In this study, fungi in the soil samples were mainly distributed among the dominant fungi in the soil, namely, Ascomycota, Basidiomycota, and Zygomycota, which is consistent with previous research results (Xu et al., 2012), and this study confirms that ascomycetes are an important phylum in continuous cropping soybean soil (Li et al., 2010). This study found that different cropping systems changed the composition and structure of Ascomycota significantly. Recent speculation suggests that changes in microbial community composition, such as a decrease in the number of soil-borne pathogens or an increase in the population of beneficial agricultural microorganisms, may be more important in disease suppression than microbial diversity (Peralta et al., 2018). Therefore, enhancing our understanding of microbial composition in different crop rotation systems is very important to determine the classified biological indicators of soil health, which is conducive to the application of effective crop rotation systems.

### The Relative Abundance of Potentially Pathogenic and Beneficial Fungi in Different Cropping Systems Was Significantly Different

Studies have shown that short-term continuous soybean cropping leads to an increase in the relative abundance of *Fusarium*, *Humicola*, and *Alternaria*. *Fusarium* and *Alternaria* are the main pathogenic fungi in soybean fields, and their abundance is significantly high during 1 and 2 years of SC (Bai et al., 2015; Cheng et al., 2017). *Humicola* was not detected in the soil samples of this study. However, SC, FS and WS promoted an increase in the relative abundance of *Fusarium* in the soil, and WS and CS promoted an increase in the relative abundance of *Alternaria* in the soil (Figure 5A). *Fusarium* is well known to be a pathogenic fungus that leads to soybean *Fusarium* root rot (Chang et al., 2018), and *Alternaria* can infect various crops and cause corresponding diseases, such as soybean Alternaria leaf spot, tomato, and carrot black rots, citrus fruit gray rot and cereal black point (Li and Yang, 2009; Logrieco et al., 2009).

There are also some potential pathogens in the soil. Research reports indicate that *Volutella* can cause diseases of other legumes (Cannon et al., 2012). Some studies have reported that *Boeremia* caused stem rot of Origanum dubium in Cyprus (Origanum dubium Boiss) and black rot of artichoke (Cynara scolymus) in California (Koike et al., 2016; Samouel et al., 2016). *Lectera*, a new genus of Plectosphaerellaceae, is a legume pathogen (Cannon et al., 2012). Studies have proven that *Bipolaris* and *Sarocladium* can cause diseases of gramineous crops (Saravanakumar et al., 2009) and maize smut disease caused by *Bipolaris* (Li et al., 2016).

In this study, FS, CS, and WS promoted an increase *in the* relative abundance of Volutella in the soil. WS promoted an increase in the relative abundance of *Boeremia* and *Bipolaris*, and WS reduced the relative abundance of *Sarocladium* in the soil. The relative abundance of *Bipolaris* in CS and SC soils was significantly low. It was found that CK treatment could reduce the relative abundance of potential pathogens in soil. Therefore, this study demonstrated that different cropping systems can regulate potential pathogens in soil.

Biological control instead of chemical control is considered to be an important method of sustainable agricultural production (Lecomte et al., 2016). In this study, some potentially beneficial fungi formed significant differences under the different treatments. *Mortierella*, *Clonostachys* and Penicillium are antagonists of pathogens. They can inhibit pathogenic *Fusarium* and control banana Fusarium wilt and soybean root rot (Pan et al., 2013; Shen et al., 2018). A previous study reported that *Hirsutella* combined with chitosan suppressed the infestation of soybean cyst nematodes in soybean roots (Mwaheb et al., 2017). It was found that *Clonostachys* is abundant in soybean continuous cropping soil (Hamid et al., 2017). This study confirmed this result and found that WS promoted a significant increase in *Clonostachys* in soil. The present results show that the relative abundance of *Penicillium* was significantly lower in CS, WS, and SC. Consistent with previous studies, continuous planting significantly reduced the relative abundance of *Penicillium* (Wu et al., 2016). The relative abundance of the beneficial fungus *Penicillium* was significantly high in CK soil.

To reveal the potential interaction between potential pathogens and beneficial fungi. Pearson correlation analysis showed that the beneficial fungus *Mortierella* was significantly negatively correlated with the potential pathogens *Alternaria* and *Boeremia*. The potentially beneficial fungus *Penicillium* was significantly negatively correlated with the potential pathogen *Alternaria* and extremely significantly negatively correlated with the potential pathogen *Boeremia*. Other potentially beneficial fungi, such as *Metarhizonium*, *Acremonium*, *Chaetomium*, and *Cryptococcus*, were significantly positively correlated with potential pathogens. The potential pathogen *Fusarium* was significantly positively related to the beneficial fungus *Metarhizoum*, which indicated that the potential beneficial fungi will increase with the increase in pathogens. However, how to control pathogens by increasing beneficial fungi in soil through crop rotation needs further research. Therefore, in future research, it is of great significance to pay attention to the interaction between members of the microbiome to regulate the function of soil microorganisms (Jiao et al., 2020).

### Fungal Communities Were Significantly Correlated With Soil Chemical Properties

Soil chemical properties were significantly correlated with soil microbial community structure (Liu et al., 2014b). In this study, the soil chemical properties of different cropping systems were significantly different, and the soil chemical properties were significantly correlated with the soil microbial community structure. The relative abundance of Basidiomycota was significantly higher in CK, and Basidiomycota was negatively correlated with the content of available Fe in the soil. The relative abundance of Zygomycota was significantly higher in FS, and there was a very significant negative correlation between Zygomycota and the content of available P in the soil. The relative abundance of Ascomycota increased significantly in WS, which was positively correlated with the content of available Cu in the soil. These results are inconsistent with previous research results (Luo et al., 2016; Cai et al., 2017), which is due to different soil regions, types and cropping systems.

Soil type and environmental factors affect the soil microbial community structure (Luo et al., 2016; Pang et al., 2017). That different cropping systems changed the relative abundance of potential beneficial fungi in the soil. There are many reasons for the change in microbial community composition. An imbalance in soil nutrients, decomposition of soil physical and chemical properties and accumulation of virulence will affect the structure of the fungal community (Wu et al., 2015). Some of them promote the production of some soil-borne pathogenic microorganisms, while others may be conducive to the production of beneficial microorganisms, resulting in changes in the number of relevant microorganisms (Li et al., 2014).

Changes in microbial community structure are related to available nutrients and plant biomass in soil (Bokhorst et al., 2017). Predecessors have shown that organic matter has an important impact on the composition of the soil fungal community (Sun et al., 2016). This study showed that the contents of OM, available Mn, available K and soil pH were significantly negatively correlated with some potential pathogenic fungi. The contents of available Fe and Zn in the soil were significantly positively correlated with potential pathogens and beneficial fungi. In this study, *Fusarium* was significantly negatively correlated with the effective content and pH of soil OM and Mn. *Penicillium* was negatively correlated with the content of available Cu and B in the soil. Predecessors have shown that cropping systems, soil chemical properties and soil microbial community structure are interrelated and interactive (Ofek-Lalzar et al., 2017). This study shows that there is a more significant correlation between soil trace elements and potential pathogens and beneficial fungi in soil. How to regulate the composition of fungal community structure in soil through trace elements and form a stable disease-inhibiting soil needs further research and proof.

## Conclusion

The fungal communities in FS, SC, WS, and CS were significantly different from those in CK. FS, SC, WS, and CS promoted the formation of fungal abundance and diversity, and different planting methods formed their own unique fungal species. FS and SC showed significantly higher fungal community abundance and species diversity than CK and CS. The abundance of *Fusarium* in FS, SC, and WS in the soil was significantly increased. CK and CS can reduce the relative abundance of *Fusarium* in soil, and CK can significantly reduce the relative abundance of many potential pathogens in soil. The potential pathogens *Alternaria*, *lectera*, and *Gibberella* were significantly positively correlated with the potentially beneficial pathogens *Mortierella*, *Chaetomium*, and *Cryptococcus*. The major potential pathogens *Alternaria*, *lectera*, *Gibberella*, and *Fusarium* in soil were significantly correlated with trace elements, and *Fusarium* was significantly negatively correlated with organic matter and Mn. Cropping system and soil physical and chemical properties are the main factors related to the formation of soil fungal structure. These results provide important significance for the selection of an effective crop rotation system and sustainable agricultural development.

## Data Availability Statement

The datasets presented in this study can be found in online repositories. The names of the repository/repositories and accession number(s) can be found below: https://www.ncbi.nlm.nih.gov/sra/, SRP164820.

## Author Contributions

XS and BT planned and arranged the entire study. XS performed the main experiments. LH, YL, and CZ assisted in the soil testing. XS and WZ wrote the manuscript together. BT participated in further discussion. All authors discussed and approved the final version of the manuscript.

## Conflict of Interest

The authors declare that the research was conducted in the absence of any commercial or financial relationships that could be construed as a potential conflict of interest.

## Publisher’s Note

All claims expressed in this article are solely those of the authors and do not necessarily represent those of their affiliated organizations, or those of the publisher, the editors and the reviewers. Any product that may be evaluated in this article, or claim that may be made by its manufacturer, is not guaranteed or endorsed by the publisher.
