# Supplementary material for: Characteristics of Soil Fungal Communities in Soybean Rotations
Source: Front Plant Sci. 2022 Jun 23;13:926731. doi: 10.3389/fpls.2022.926731 (PMC9260669; doi:10.3389/fpls.2022.926731)
Supplement: Supplementary file 1 [file Table_1.DOCX]

**Supplementary Figure 1**. Cluster analysis of similarity of different cropping systems. SC: soybean continuous cropping; CK: fallow; FS: fallow-soybean; CS: corn–soybean; WS: wheat–soybean.

**Supplementary Figure 2**. Genus of fungi with significant differences between SC and CK soils. * indicates a significant correlation (*P* < 0.05), * * indicates an extremely significant correlation (*P* < 0.01).

**Supplementary Figure 3**. Genus of fungi with significant differences between SC and FS soils. * indicates a significant correlation (*P* < 0.05), * * indicates an extremely significant correlation (*P* < 0.01).

**Supplementary Figure 4**. Genus of fungi with significant differences between SC and CS soils. * indicates a significant correlation (*P* < 0.05), * * indicates an extremely significant correlation (*P* < 0.01).

**Supplementary Figure 5**. Genus of fungi with significant differences between SC and WS soils. * indicates a significant correlation (*P* < 0.05), * * indicates an extremely significant correlation (*P* < 0.01).

**Supplementary Figure 6.** Relative abundance of potentially pathogenic and beneficial fungi in soils with different cropping systems. (**A)** Relative abundance of potential pathogenic genera in soil under different cropping systems; (**B)** Relative abundance of potentially beneficial genera in soils under different cropping systems. There was a significant difference in the representation of different letters (*P* < 0.05)

**Supplementary Table 1.** Quality evaluation of sequencing data.

| Sample | Length Filter (100 bp) | Clean | %Clean | %Q20 | %Q30 |
| --- | --- | --- | --- | --- | --- |
| CK-1 | 235935 | 221491 | 93.88% | 99.50% | 97.15% |
| CK-2 | 122692 | 111386 | 90.79% | 99.35% | 96.33% |
| CK-3 | 324156 | 304158 | 93.83% | 99.54% | 97.35% |
| FS-1 | 358965 | 338061 | 94.18% | 99.55% | 97.32% |
| FS-2 | 332478 | 313168 | 94.19% | 99.51% | 97.07% |
| FS-3 | 226326 | 212055 | 93.69% | 99.48% | 96.95% |
| CS-1 | 178581 | 170437 | 95.44% | 99.59% | 97.44% |
| CS-2 | 327697 | 311197 | 94.96% | 99.63% | 97.66% |
| CS-3 | 448433 | 426619 | 95.14% | 99.60% | 97.50% |
| WS-1 | 186718 | 177479 | 95.05% | 99.59% | 97.45% |
| WS-2 | 214625 | 203108 | 94.63% | 99.55% | 97.19% |
| WS-3 | 278491 | 264420 | 94.95% | 99.58% | 97.39% |
| SC-1 | 206630 | 195676 | 94.70% | 99.57% | 97.33% |
| SC-2 | 207356 | 196165 | 94.60% | 99.57% | 97.34% |
| SC-3 | 328455 | 310431 | 94.51% | 99.51% | 97.03% |

Note: The values of Q20 (%) and Q30 (%) represent 1% and 1‰ of base errors, respectively.

**Supplementary Table 2.** Statistical table of optimization sequence.

| Total number of samples | Number of sequences | Total residues | Average length（bp） |
| --- | --- | --- | --- |
| 15 | 3755851 | 925431395 | 246.4 |

**Supplementary Table 3.** Effects of different cropping systems on the composition of microbial communities of several fungal phyla in soil.

|  | CK | | FS | CS | WS | SC |
| --- | --- | --- | --- | --- | --- | --- |
| Ascomycota | | 15.79 ± 1.56^d^ | 55.51 ± 3.12^c^ | 70.36 ± 3.40^b^ | 79.40 ± 1.62^a^ | 69.16 ± 3.13^b^ |
| Basidiomycota | | 65.98 ± 5.37^a^ | 17.78 ± 3.59^b^ | 20.78 ± 3.47^b^ | 9.69 ± 0.58^b^ | 14.19 ± 4.47^b^ |
| Zygomycota | | 16.29 ± 3.66^ab^ | 19.78 ± 5.46^a^ | 6.13 ± 0.34^b^ | 6.16 ± 0.94^b^ | 9.17 ± 1.29^b^ |
| No_Rank | | 1.74 ± 0.16^e^ | 6.11 ± 0.13^b^ | 2.52 ± 0.20^d^ | 4.33 ± 0.14^c^ | 6.71 ± 0.06^a^ |
| Glomeromycota | | 0.00 ± 0.00^d^ | 0.44 ± 0.09^a^ | 0.16 ± 0.05^c^ | 0.36 ± 0.04^ab^ | 0.22 ± 0.00^bc^ |
| Chytridiomycota | | 0.03 ±0.02^a^ | 0.35 ± 0.26^a^ | 0.03 ± 0.01^a^ | 0.05 ± 0.01^a^ | 0.01 ± 0.00^a^ |
| Rozellomycota | | 0.16 ± 0.03^a^ | 0 | 0 | 0 | 0 |
| Cercozoa | | 0.01 ± 0.00^b^ | 0.04 ± 0.01^a^ | 0.01 ± 0.00^b^ | 0.01 ± 0.00^b^ | 0.01 ± 0.00^b^ |

Note: the figures in the table represent the mean ± standard deviation (n = 3). For each column, different letters represent significant differences (*P* < 0.05).

**Supplementary Table 4**. Relative abundance of the genera of dominant fungi in soil under different cropping systems.

|  | Alternaria (%) | Chaetomium (%) | Tetracladium (%) | Gibberella (%) | Peziza (%) | Lectera (%) | Hydropisphaera (%) | Mortierella (%) | Sistotrema (%) | Cortinarius (%) | Amanita (%) |
| --- | --- | --- | --- | --- | --- | --- | --- | --- | --- | --- | --- |
| CK | 0.39 ± 0.18b | 0.30± 0.15b | 0.43±0.07d | 0.05±0.01c | 0.01±0.00c | 0.01±0.00b | 0.00±0.00b | 15.66±3.59ab | 0.02±0.02b | 16.66±3.14 a | 13.00±2.61a |
| FS | 2.95±0.19b | 1.02± 0.21b | 2.95±0.10c | 2.93±0.50b | 8.01±0.84a | 0.07±0.03b | 0.36±0.10b | 18.28±5.38 a | 2.44±0.68ab | 0.08±0.05b | 0.01±0.01b |
| CS | 10.65 ± 2.00a | 8.23± 1.76a | 4.97±0.1b | 5.58±0.91a | 0.48±0.06c | 0.47±0.10b | 4.52±1.04a | 6.09±0.34 b | 12.01±5.34a | 0.00±0.00b | 0.00 ±0.00b |
| WS | 11.45 ± 1.39a | 6.21± 0.80a | 3.62±0.34c | 2.15±0.29c | 1.09±0.15c | 4.95±2.64ab | 6.07±0.77a | 6.10±0.93 b | 1.16±0.22b | 0.00±0.00b | 0.00±0.00b |
| SC | 3.87 ± 0.62b | 7.57± 1.67a | 7.32±0.82a | 5.67±1.80a | 5.94±0.21b | 7.93±3.27a | 1.63±0.25b | 8.90±1.23 ab | 4.84±4.33ab | 0.01±0.00b | 0.01±0.00b |

Note: the figures in the table represent the mean ± standard deviation (n = 3). For each column, different letters represent significant differences (*P* < 0.05).

**Supplementary Table 5**. Correlation between microbial communities and soil fertility.

| Environmental factor  combination | Size correlation |
| --- | --- |
|  | fungi |
| Fe | 0.64 |
| Fe Mn | 0.73 |
| Cu Fe Mn | 0.81 |
| K Cu Fe Mn | 0.77 |
| K pH Cu Fe Mn | 0.75 |
| K pH Cu Zn Fe Mn | 0.74 |
| K OM pH Cu Zn Fe Mn | 0.71 |
| P K OM pH Cu Zn Fe Mn | 0.69 |
| N P K OM pH Cu Zn Fe Mn | 0.64 |
| N P K OM pH Cu Zn Fe Mn B | 0.58 |

Note: the number in the table indicates a significant correlation (*P* < 0.05).
