# Supplementary figures and images for: Characteristics of Soil Fungal Communities in Soybean Rotations
Source: Front Plant Sci. 2022 Jun 23;13:926731. doi: 10.3389/fpls.2022.926731 (PMC9260669; doi:10.3389/fpls.2022.926731)

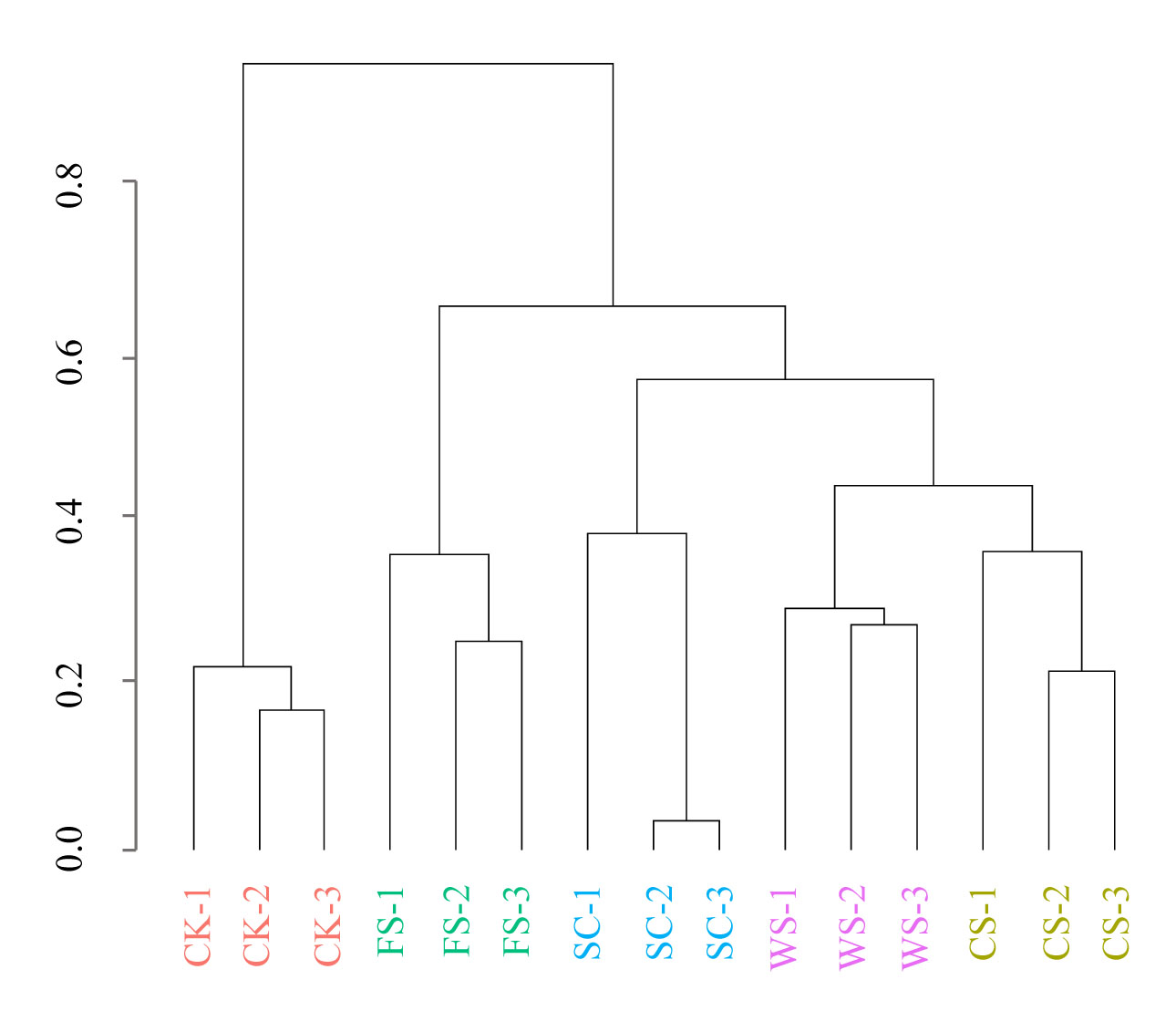

Supplement: Supplementary file 2 [file Image_1.JPEG]

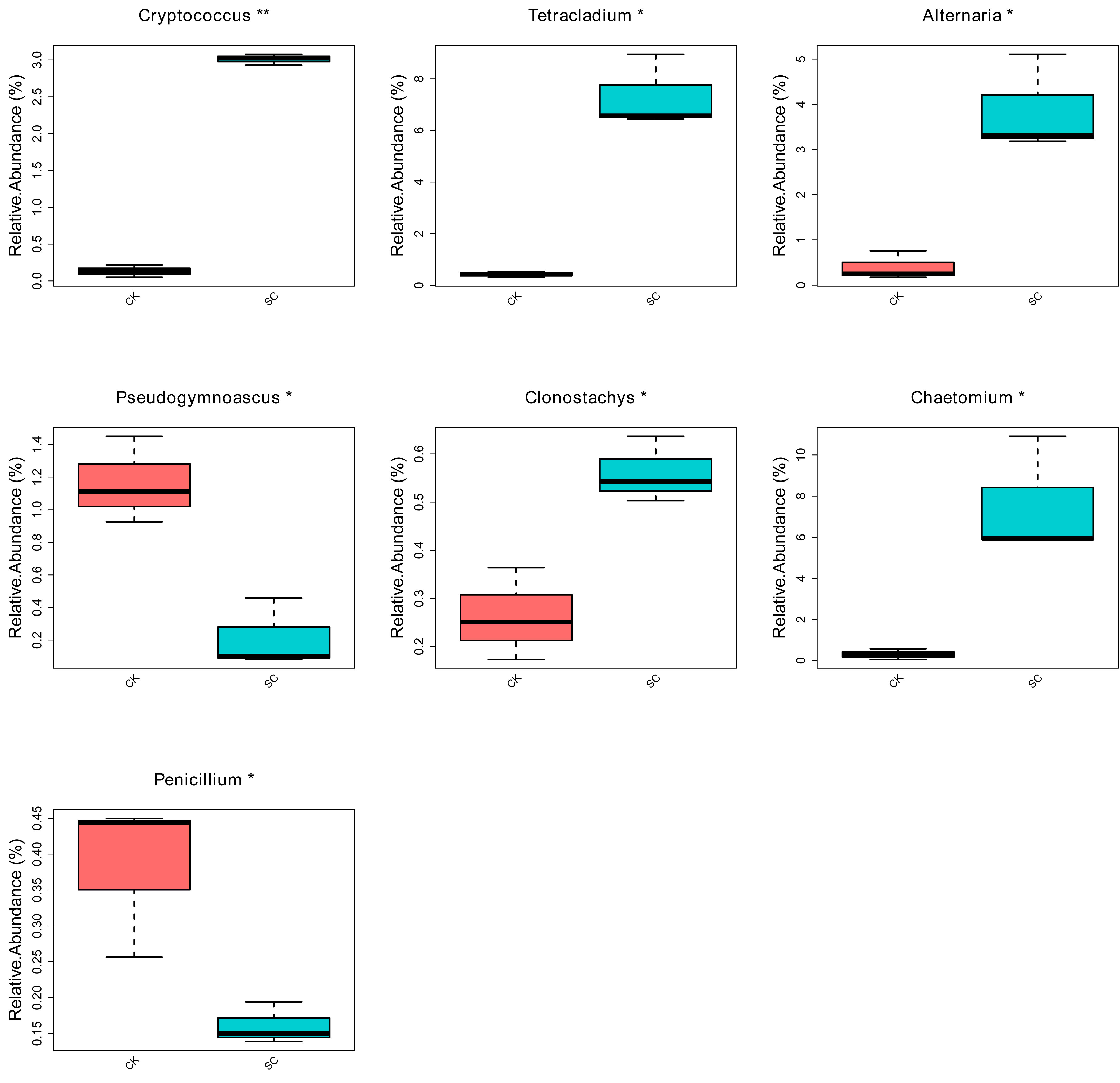

Supplement: Supplementary file 3 [file Image_2.JPEG]

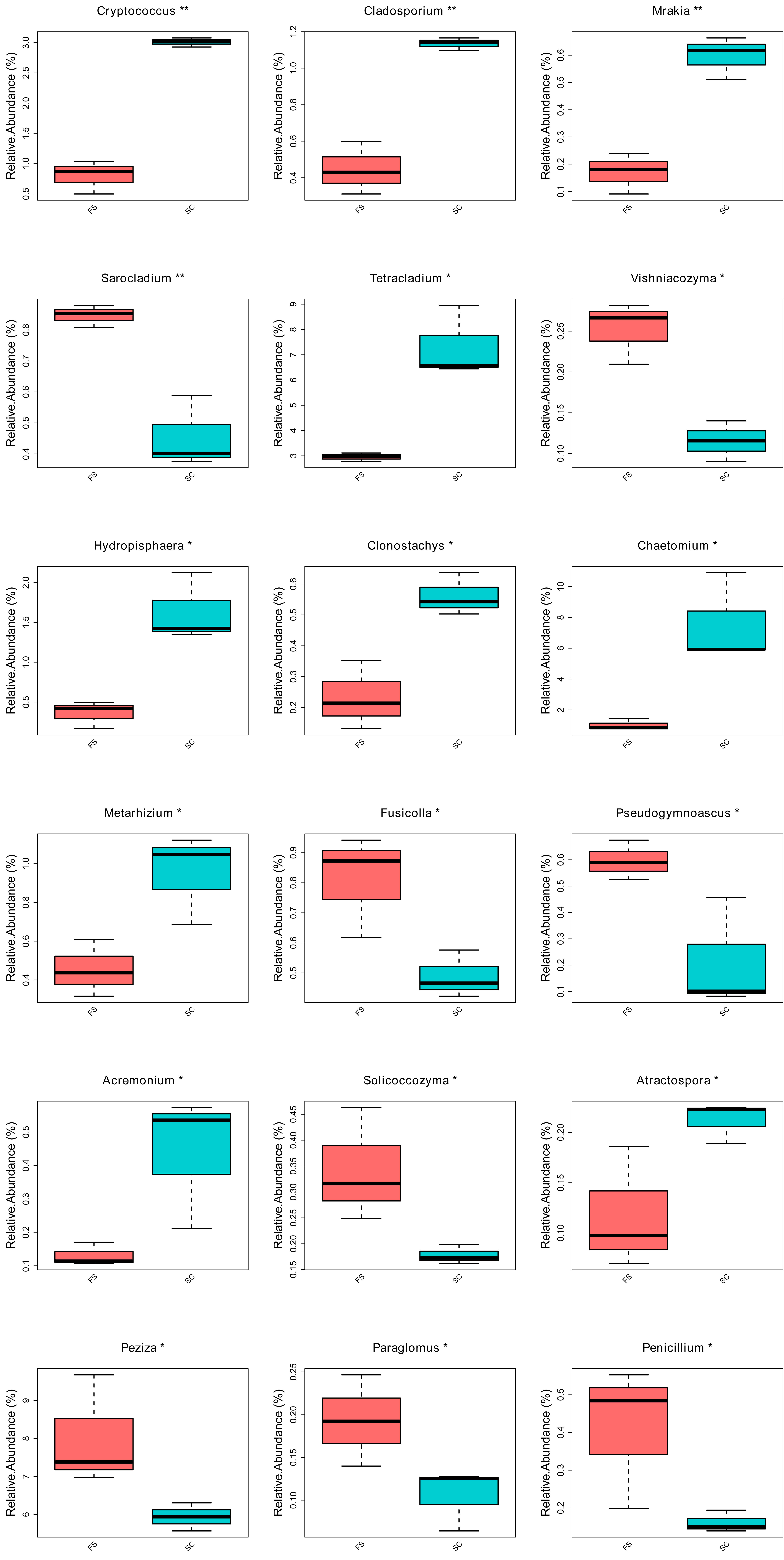

Supplement: Supplementary file 4 [file Image_3.JPEG]

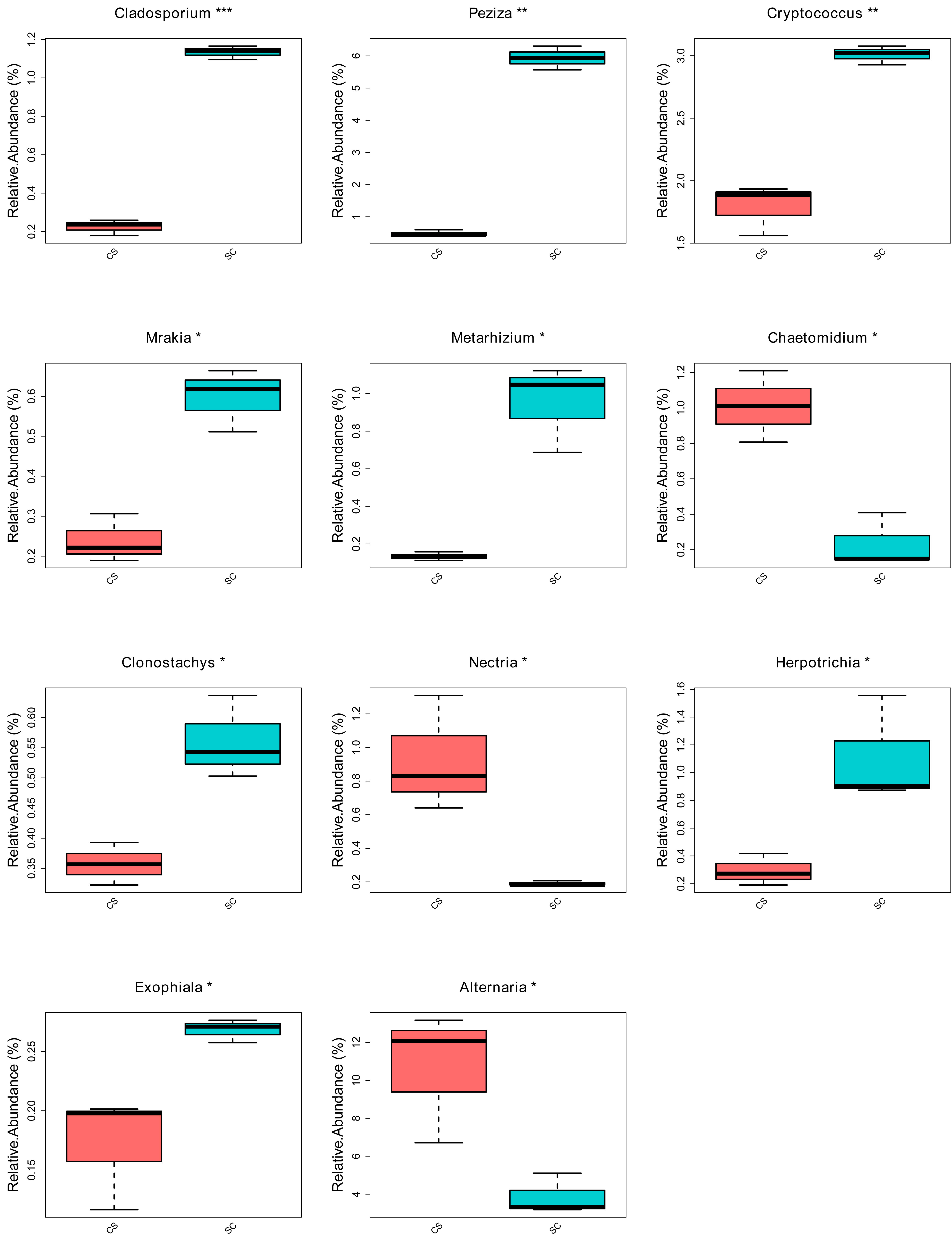

Supplement: Supplementary file 5 [file Image_4.JPEG]

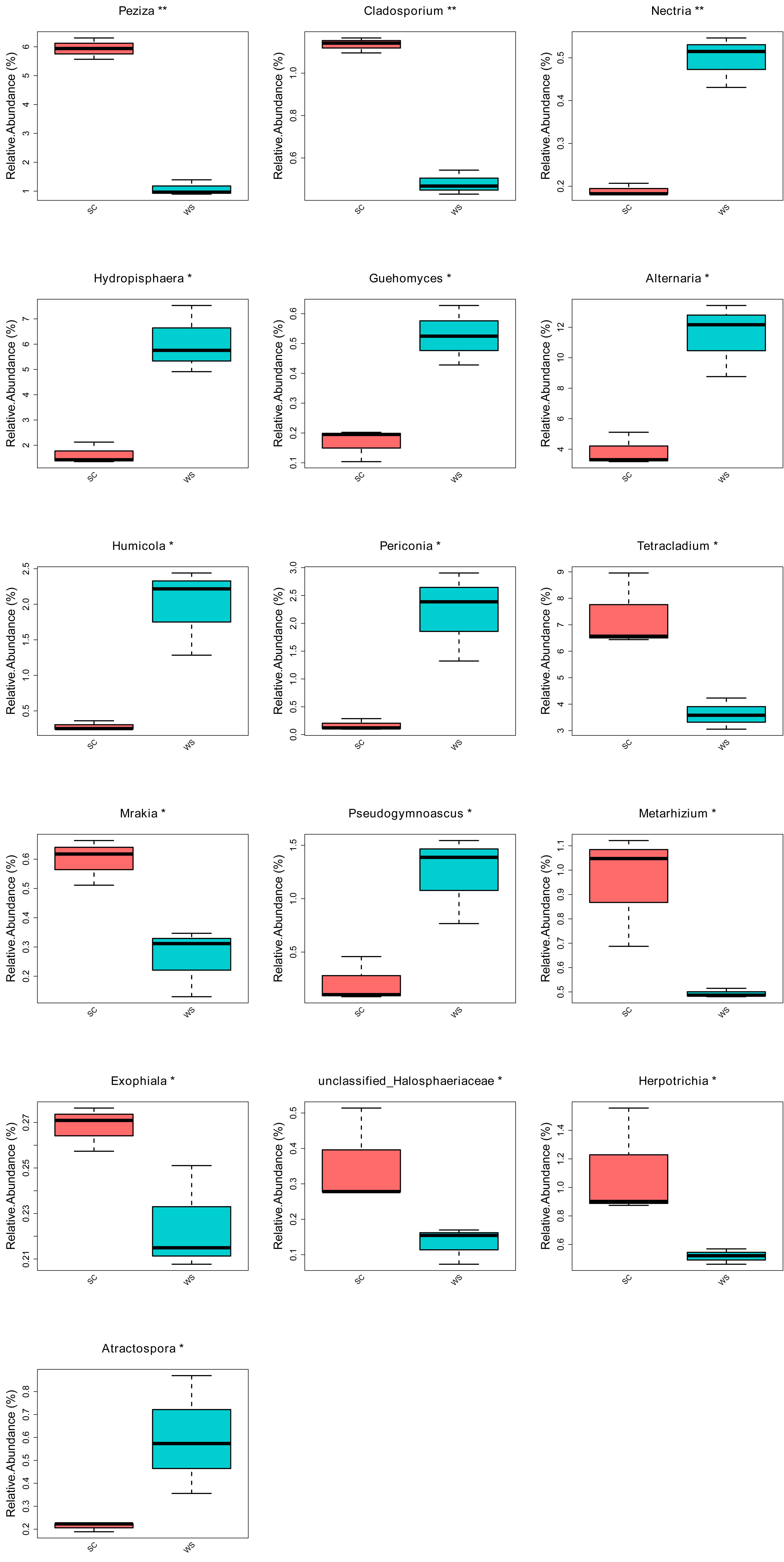

Supplement: Supplementary file 6 [file Image_5.JPEG]

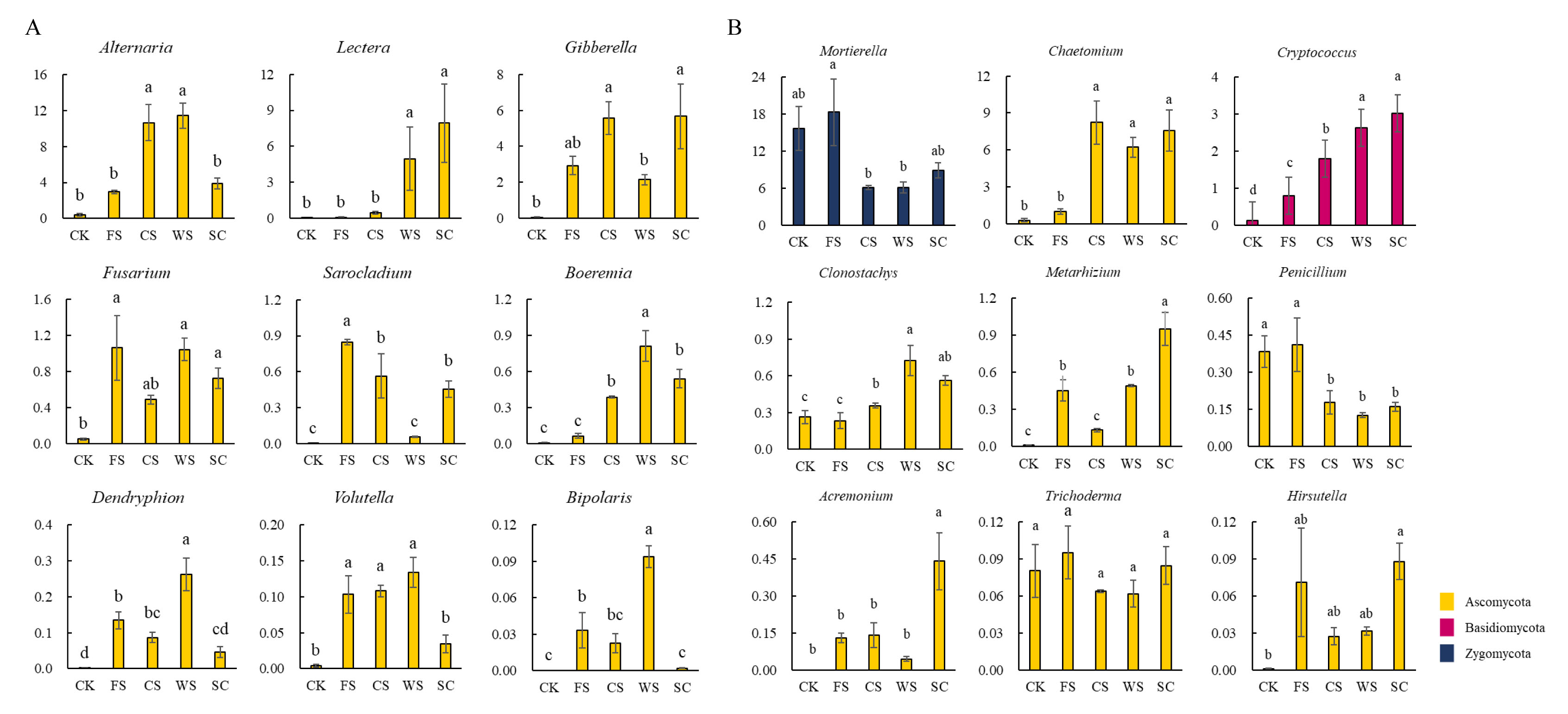

Supplement: Supplementary file 7 [file Image_6.JPEG]
